# Learning new sport actions: Pilot study to investigate the imitative and the verbal instructive teaching methods in motor education

**DOI:** 10.1371/journal.pone.0237697

**Published:** 2020-08-14

**Authors:** Elisa De Stefani, Francesca Rodà, Elio Volta, Vincenzo Pincolini, Andrea Farnese, Stefano Rossetti, Federica Pedretti, Pier Francesco Ferrari

**Affiliations:** 1 Department of Medicine and Surgery, University of Parma, Parma, Italy; 2 Giocampus Steering Committee, Parma, Italy; 3 Institut des Sciences Cognitives Marc Jeannerod, CNRS, Université de Lyon, Bron, France; Universita degli Studi di Udine, ITALY

## Abstract

The aim of the project was to investigate the effects of two strategies of teaching new sport actions on performance of eight-year-old children: observational-imitative method (OIM) and descriptive-directive method (DDM). The OIM group was provided with a pre-practice instruction in the form of expert modeling observation by an expert athlete. The DDM group received only verbal explanations of few selected static images. Thirty-six children (18 males and 18 females, mean age = 8,8) participated in the experiment. Subjects were randomly assigned to the OIM or DDM groups. Participants were instructed to perform four sport motor sequences never performed before (shoulder stand, soccer action, vortex howler throw, step action). Actions were videotaped and 2D kinematic analysis performed. A 10-point Likert questionnaire was administered to blind sport experts to assess the correctness and accuracy of each action. Results suggest that the OIM is the most effective instruction method when participants have no experience with the sport action to be performed. On the contrary, if the athlete needs to learn specific aspects of an exercise (such as grasping a tool) the best method is the DDM. In fact, detailed information on how to grab the vortex helped children in throwing it. We also found gender differences which might reflect cultural influences in specific sports (e.g. soccer). Finally, repetition of the exercise also improved the DDM group’s performance. This has potential applications in sport teaching, suggesting that in the absence of a model performing the action to be imitated, the DDM can be as effective as the OIM if the observer repeats the sport action many times.

## Introduction

Motor learning is generally defined as a set of processes aimed at learning new skills by practice. Haibach-Beach et al. claimed that “*motor learning is a relatively permanent change in the ability to execute a motor skill as a result of practice or experience*”[1]. Practice facilitates the development of motor skill for movement production that would enable the performer to generate new movements [1]. Skill learning typically involves the generation of a new movement pattern and the formation of this motor pattern is favored by the recognition of similarities with skills already present in the own motor repertoire [2,3]. Each new motor patterns would result in a perceptual trace, and the repetitions of the movements permit the accumulation and reinforcement of perceptual traces in memory [2,3]. One way to acquire new movements is to observe a model and imitate his behavior. One body of research suggest that model observation can significantly speed up the learning process, reduce the amount of trial-and-errors that are needed to accomplish the movement goal and provide a correct example of a successful performance [4]. According to simulation theories, cortical motor simulation is fundamental to imitate the actions of others [5,6]. One of the core neural underpinnings of such cognitive skills is represented by mirror neuron mechanisms (MNM), which are capable of activating motor representations in parietal and premotor cortical regions while an individual is observing a motor act. Such correspondence between perceived and executed actions has been proposed as the neural substrate of cortical motor simulation [7–10]. Several theoretical and experimental studies have further expanded the functional role of MNM, and have proposed that the temporary activation of specific motor programs during the observation of an action might be exploited by the observer in order to understand, respond and imitate the observed action [8,10–16]. In Calvo-Merino et al’s study [17], the authors used fMRI to investigate how motor familiarity effects neural responses during sport action observation in expert dancers. Expert athletes showed increased activity in mirror neuron mechanisms areas while watching movements from their own discipline compared to other dance disciplines. This means we can activate a mental image of an observed movement because it is in our own motor repertoire, and this facilitates its reproduction. MNM are activated not only for familiar actions, but also during the learning of a new action sequences [8,18] allowing an observer to replicate the action presented by a model [12,19].

In the domain of sports and physical education, coaches and teachers have always searched for ways of being more efficient in their teaching and observational practice was used in sports settings to enhance motor skill [20,21]. Several studies have reported how the physical performance of baseball [22], basket [23], gymnastics [24], or tennis athletes [25] was enhanced after action observation training. Moreover, learning by observing was better than motor imagery training during the early phase of motor learning [26]. Especially at the beginning of the imitation process, it is necessary to extract meaningful features from the model’s action. A number of studies examined instructions and feedback for motor skill learning and their influence on motor performance. In a recent study [27] authors aimed at disentangling visual and verbal instruction modalities in dance movements. They found a performance superiority of visual observation in learning of complex movement sequences in dance. Similar results were observed for learning the golf swing [28], volleyball [29] and jump-landing task [30]. These results indicated observational practice as a powerful source of information that improves the performance of a motor skill [31–33]. Instructions directed at the movement outcome (external focus of attention) are also more effective than those directed at the performer’s body movements (internal focus, [34,35]).

Even though model observation is considered to be one of the most important methods for learning new skills in both children and adults [36,37], relatively little is known about how an imitation learning may be utilized most efficiently during development [38].

For example, it is not known whether, in the initial learning of a new action, a verbal explanation of the movements to be performed could be more effective than a model observation [32]. From the developmental perspective several studies have shown that the development of motor control is tied to the ability to integrate visual and proprioceptive afferences between 5 to 10 years of age, during the execution of overt movement [25–27]. Children less than 8–9 years of age [28] predominantly use visual feedback to correct their movement. Only around 9 years is there a shift from the use of visual to proprioceptive information for correcting movement. Although imitation permits the most spontaneous way to learn sport actions [39], in the absence of mature proprioceptive abilities to correct movements, the goal to be achieved (e.g. kicking a ball) can prevent the correct execution of the basic motor sequences necessary to correctly perform the action. Consequently, the acquisition of new skills is limited in the absence of additional cues from the teacher/instructor. Previous research demonstrated that a visual model is not sufficient for facilitating motor performance in elementary-aged children and the use of verbal rehearsal in the instructions resulted in better performance [40]. Furthermore, studies on imitation in children demonstrate that preschoolers represent the most salient goal of the action to be imitated, but often ignore the sub-goals that are embedded in the action sequence [41]. Therefore, preschoolers will emulate the action (e.g. kicking a ball), but ignore the whole sequence of motor acts and sub-goals present in the whole action sequence (e.g. take the ball with your hands, throw it in the air, let it bounce on the ground and then kick it). Consequently, for a sport action that requires attention to detail, the parsing of the sequence into the sub-goals of each motor task is critical for the achievement of a new motor skill [42]. Under these circumstances, in order to teach new movements that are not within an individual’s action repertoire, it is typical to try and focus a child’s attention on specific features of the movement using verbal instruction. It is still not clear what information is perceived and used for initial movement reproduction [43] and if the explanation by the instructor is not clear, the children might not fully understand the main features of a new action sequence and the best performance may not be reached [44].

The lack of specific guidelines leads instructors and education teachers to autonomously decide on the details of the training protocol, the practice duration, with a methodological approach which might be not suitable for children at a specific age. For this reason, in the current study we investigated two different teaching strategies in order to elucidate whether one is more efficient for the acquisition of new sport actions in primary-school children. Specifically, we wanted to determine whether providing specific instructions focused on keys aspects of an action would improve the performance of participants. To achieve this aim, we compared: 1) the observational-imitative method (OIM) of instruction; and 2) the verbal-instruction method (descriptive-directive method; DDM). In the case of the OIM, a group of children were provided with pre-practice video instructions in the form of modeling observation by an expert athlete, in accordance with the extensive MNM literature [8,12,14]. In the case of the DDM, a different group of children were given verbal instructions and a few static photos of the action to be performed, with a couple of studies suggesting this to be an effective teaching method for very young children [45,46]. 3) We also considered how the method of instruction may influence the performance of males and females differently. A large number of studies report sex differences in language performance: females appear superior to males in terms of verbal and linguistic skills, from infancy through to adulthood [47–50]. Therefore, we expected a better performance of females than males in the DDM group. Finally, 4) we also examined whether the consecutive repetition of a new action sequence improved performance in both OIM and DDM groups. Schmidt [2] pointed out that the number of repetitions of a gesture to be learned is fundamental to form and strengthen an action scheme and a large amount of literature claimed that observational practice (particularly when it is combined with physical practice), can make important contributions to learning (for a review see [34]).

All sport exercises were performed and videotaped in the gym were children are used to play sport, with the aim to preserve an ecological context. The 2D kinematics of each sport action was analyzed offline. In addition, the performance of the children was judged by a group of sports experts who assessed the quality (in terms of speed, fluidity, accuracy of the movement) of the performed actions (see S1 Materials).

## Materials and methods

### Participants

Thirty-six children participated to the experimental study (18 males and 18 females, M_age_ = 8,8 ±0.73) and they were randomized equally to two groups, OIM and DDM, stratified by gender. A statistical power analysis for sample size estimation was previously performed (GPower 3.1.9.2): Test Family F tests and Statistical Test ANOVA: Repeated Measure, within–between interaction. The parameters used were: α = 0.05 (Two Tailed); 1-β = 0.95; number of repetitions = 2; groups = 2; supposed correlation among measures = 0.5. The effect size f considered was based on results of the feasibility study (see S1 Materials: main group effect: η^2^ = 0.33 corresponding to Effect Size f = 0.70; Interaction group*gender*task: η^2^ = 0.22 corresponding to Effect Size f = 0.53) and was f = 0.53. The projected sample size needed with this effect size is N = 36 (n.18 for each group). A subject was discarded in shoulder stand and soccer actions because technical problems. Exclusion criteria were: (1) the achievement of a score < = 70 percentile (M = 30.6; SD = 3.13) at the Test Coloured Progressive Matrices, CPM (a test used for the evaluation of cognitive functions, general components of fluid intelligence which allows the investigation of the capacity of problem- solving; [51]); (2) any orthopedic or neurological problems that would interfere with the ability to perform a coordinated arm movement; (3) left dominant hand. Informed consent and assent for participation in the study were obtained from children and their parents/legal guardians. The Ethical Committee of the University of Parma approved the study.

### Stimuli

**Fig 1:**
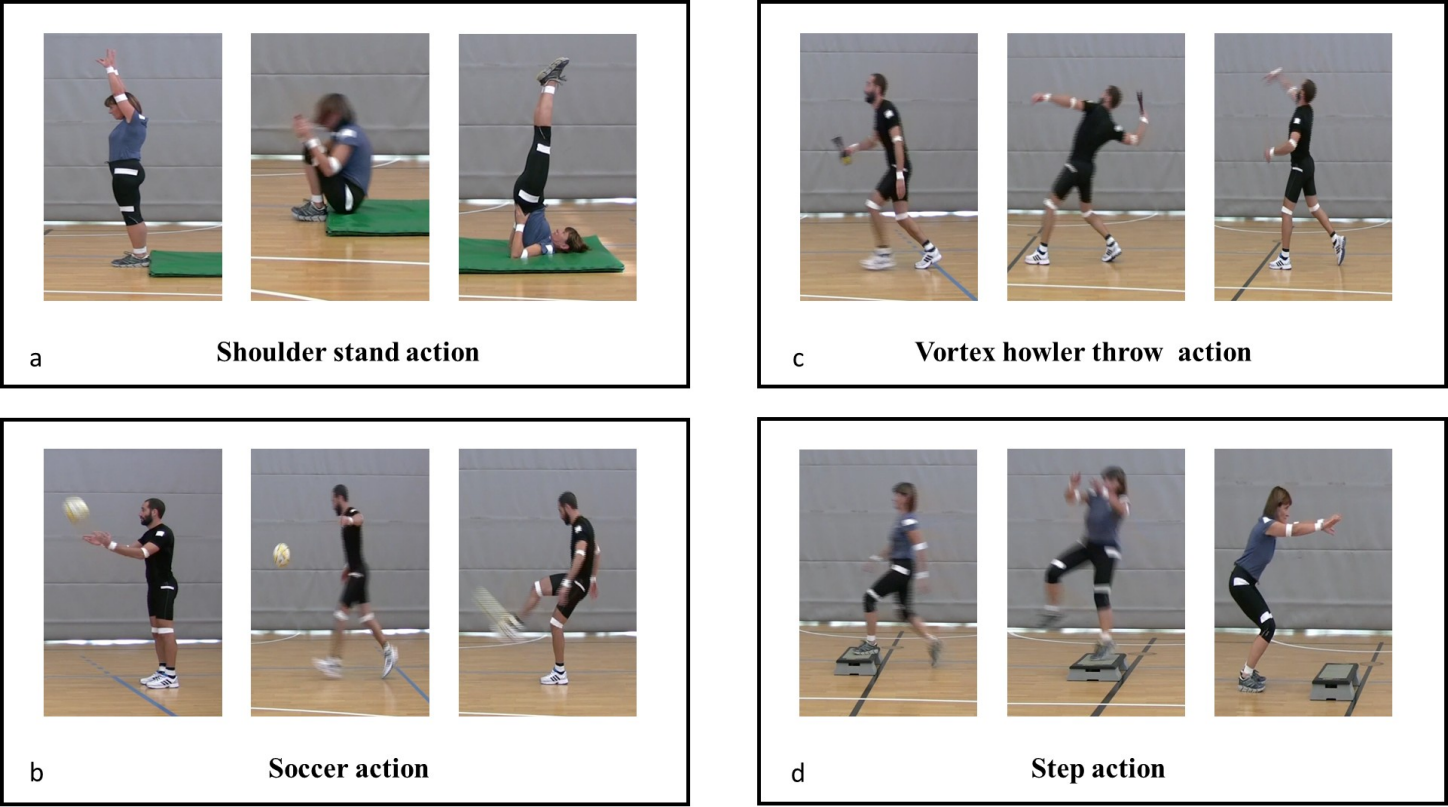
Sport actions investigated in the project. The Figure is representing the four actions investigated, showing the three main moments of each: Starting, intermediate and final phase.

The sport actions selected for use here were as follows: shoulder stand, soccer action, vortex howler throw, step action (Fig 1). The sport actions were chosen by several consultants, including physical education teachers, professional association and sporting organization representatives, coaches and university lecturers belonging to Giocampus project (Parma). The consultants were invited to participate in a consensus form process to assist in the selection of skills to be included.

In the soccer action, the ball was initially thrown with both hands and then kicked with the right instep after the first rebound.

The shoulder stand is an inversion that starts by lying down on the floor. Children had to bend the knees and place the feet on the floor as close to the buttocks as possible. They then had to lift their legs up overhead, detaching their hips and legs from the mat perpendicular to the floor. Once balance was reached, with hands on the hips, they had to maintain the position for five seconds.

In the Vortex howler throw, after a short run-up, participants had to reach a standing position with both feet behind the throwing line and throw the tool parabolically. The parabolic trajectory required maximum arm extension.

In the step action, the participants performed a short run up. Then they were required to jump the step on one foot, twist their body 180°, falling back on the ground with both legs bent and feet parallel. All videos and instructions are in the S1 Materials. All children reported having kicked or thrown an object or jumped an aerobic step prior to the study. None of them had ever performed the shoulder stand action, nor grabbed a vortex.

### Procedure

Participants were instructed to perform each action sequence seven times (shoulder stand, soccer action, vortex howler throw, step action), according to the assigned instruction (OIM or DDM). Depending on the group the children had been assigned to the individual participant’s session started with OIM or MDD learning. The OIM group observed three minutes of videos in which an athlete performed the sport actions. No verbal explanation was given. The MDD group was given three minutes of verbal instructions and a few static photos (4–5 images) extracted by the OIM videos describing specific phases of the action to perform. Verbal cueing followed each photo. Ten national sport experts selected which key frames of each action were to be extracted and verbal instructions to give to children (Fig 1). After receiving the OIM or MDD instructions, the children performed each action seven times. Participants were also instructed to perform the movement within a space delimited by markers marked on the floor (Fig 1). Each trial was initiated by a “go” signal given by the experimenter. There was a rest interval of 2 min following every action. All participants were tested individually in the presence of the experimenter only.

Anatomical landmarks (see Fig 1) were attached to participants with scotch tape on various joints (shoulder, wrist, knee, ankle) and used to reconstruct 2D movements. Children’s performances were videotaped. The sport experts evaluated the correctness of each videotaped sport actions. A 10-point Likert questionnaire was individually administered to the experts (for further details see S1 Materials), who were unrelated to the experimental procedure and were blind to group assignment. The overall performance score given to participants allowed us to determine the most effective type of instruction for each action.

### Movement recording system and movement reconstruction

The setting was structured in order to define the space of action equally for each participant (Fig 1). For the soccer action, vortex howler throws and step action, two position markers were placed on the floor designating a start point and an end point (start position: blue line; end position: black line, Fig 1). The start position of the shoulder stand action was marked by the edge of the mat and the end position was the maximal leg extension towards the ceiling.

The sports actions were recorded from a left lateral view using a digital camera (Canon Legria HF R706), and subsequently analyzed off-line. Using software for 2D kinematic analysis (Tracker Video Analysis and Modeling Tool, 4.11.0), the first and the last repetition of each action were analyzed. Tracker Video Analysis permits to "mark" video frames, set the origin of the movement, and calibrate the video for real-world measurement values. Anatomical landmarks (see Fig 1) were attached to participants with scotch tape on various joints (shoulder, wrist, knee, ankle). Each video has been carefully inspected and the ankle marker was selected and used for the reconstruction of the movement because it provided the most stable track. Positions of the ankle marker in each frame was manually identified and followed frame by frame for a time interval (25 fps). Its y and x coordinates were recorded as a function of time. The program returns frame by frame the x and y coordinates of the selected marker and instantaneous velocity and acceleration indices. From these values we have extracted the peak of velocity and acceleration and calculated the parameters of distance. Specifically, X-Y Distances were the Euclidian distances between the starting and ending points for each task. The Norm_Path was computed with the following formula Σ(√(x_1i_-x_2i_)^2^+ (y_1i_-y_2i_) ^2^) where x = (x_1_, x_2_,…, x_ni_) and y = (y_1_, y_2_,…, y_ni_) were two points in Euclidean space between which the Euclidean distance (x-y vectors) is given by the Pythagorean formula. The kinematic parameters related to distance were normalized on each participant’s left leg length and coded adding “Norm_” prefix. Thus, the following parameters were considered for analysis: Norm_Path (cm), Norm_Distance (cm), Norm_X_Distance (cm), Norm_Y_Distance (cm), Max_PeakVelocity (mm/s) and Max_PeakAcceleration (mm/s2).

### Statistical analysis

For the first and last repetition of each sport action, the following kinematic parameters were considered as dependent measures: Norm_Path, Norm_Distance, Norm_X_Distance, Norm_Y_Distance, Max_PeakVelocity, Max_PeakAcceleration.

For each mean value of the dependent measure, a mixed-design repeated measures ANOVA was carried out with repetition of the exercise (first vs last) as a within-subject factor, and group (type of instruction OIM vs DDM) and gender (female vs males) as between-subject factors [52–54]. In all analyses, post hoc comparisons were performed using the Newman–Keuls procedure. The significance level was fixed at p = 0.05. Sphericity of the data was checked for before performing statistical analyses (Mauchly’s test, p > 0.05). η^2^ was also calculated.

To assure the internal consistency of the qualitative judgments, the Cronbach’s Alpha Test was performed, with results of 0.70 and above considered as good, 0.80 as better than good, and 0.90 and above as the best. The reliability analyses showed consistent results related to the qualitative judgments: α di Cronbach test: Shoulder stand = 0.991; Soccer = 0.980; Step = 0.973; Vortex Howler Throw = 0.977. The overall scores (Q5, see S1 Materials) attributed to the two groups by the experts are shown in the Table 2.

## Results

### Shoulder stand action

**Fig 2:**
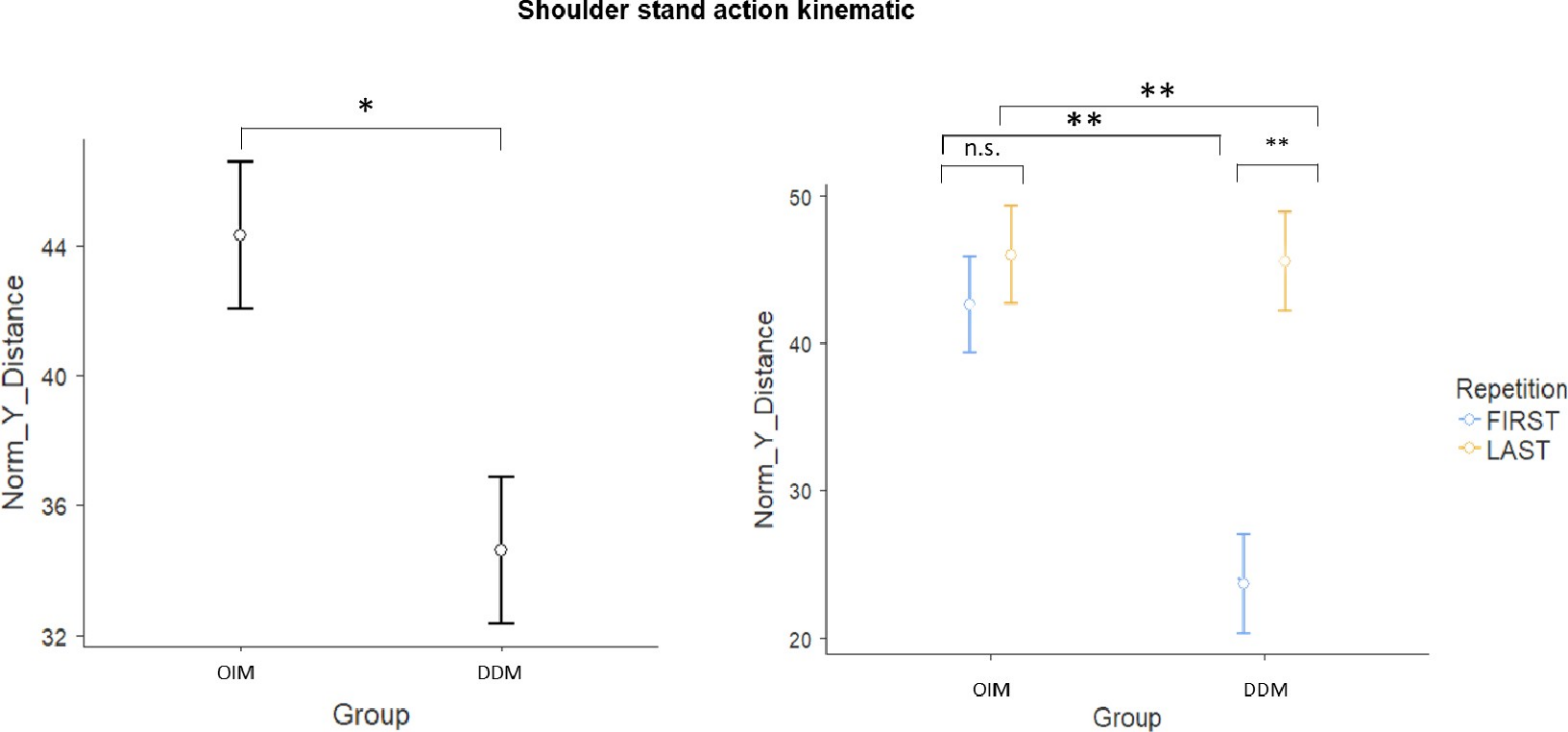
Shoulder stand action Kinematic. Main group effect (left graphic) and Group*Repetition effect (right graphic) related to the Norm_Y_Distance Kinematic parameter. OIM = observational-imitative method; DDM = descriptive-directive method; * = p<0.05; ** = p<0.01 and n.s = no statistical significance.

Considering that the shoulder stand action rises in height, following the suggestion of the experts, the most representative kinematic parameter was Norm_Y_Distance. A main group effect was found for Norm_Y_Distance (F_(1,31)_ = 9.05; p<0.005, η^2^ = 0.226), with the OIM group showing a higher leg height in the Y axis (44 ±14) then the DDM group (35 ±17, Fig 2). A group by repetition effect was also found for Norm_Y_Distance (F_(1,31)_ = 7.18; p = 0.01, η^2^ = 0.19), with the maximum leg height in the Y axis lower in the DDM group in the first repetition (24 ±18) compared to the last one (46 ±5), and to the MOI group (first = 42 ±16; last = 46 ±12 Fig 2).

Statistics of all the other kinematic parameters are reported in Table 1.

**Table 1:** Summary representation of all statistically significant results of the project.

Kinematic parameters	Shoulder Stand	Soccer	Vortex
Norm_Path	Gender Effect F_(1,31)_ = 5.109; p = 0.031, η^2^ = 0.141 Repetition Effect F_(1,31)_ = 4.153; p<0.05, η^2^ = 0.30	Group Effect F_(1,31)_ = 5.13; p = 0.031, η^2^ = 0.142	Repetition Effect F_(1,32)_ = 6.46; p = 0.016, η^2^ = 0.168
Norm_Distance	Group * Repetition F_(1,31)_ = 4.6; p = 0.04, η^2^ = 0.13	No Significant Effects	Repetition Effect F_(1,32)_ = 6.44; p = 0.016, η^2^ = 0.168
Norm_X_Distance	Repetition Effect F_(1,31) =_ = 10,8; p<0.003, η^2^ = 0.26	Group * Gender Effect F_(1,31) =_ = 5.11; p = 0.031, η^2^ = 0.142	Repetition Effect F_(1,32)_ = 5.29; p = 0.028, η^2^ = 0.142
Norm_Y_Distance	Group Effect F_(1,31) =_ = 9.05; p<0.005, η^2^ = 0.226 Group * Repetition Effect F_(1,31)_ = 7.18; p = 0.01, η^2^ = 0.19	No Significant Effects	No Significant Effects
Max_PeakVelocity	No Significant Effects	Gender Effect F_(1,31) =_ = 4.04; p = 0.053, η^2^ = 0.115	No Significant Effects
Max_PeakAcceleration	Repetition Effect F_(1,31) =_ = 6,37; p<0.017, η^2^ = 0.17	Repetition Effect F_(1,31) =_ = 4.98; p = 0.033, η^2^ = 0.138	Group Effect F_(1,32)_ = 4.44; p = 0.043, η^2^ = 0.122

All significant results are shown in the table

For this actions, the experts assigned higher scores to the OIM group (4.7±2.2) than to the DDM group (4.5±1.8; Table 2).

**Table 2:** The overall scores that the experts assigned to the actions (question Q5).

Actions	Group	Q5 Mean (± SD)
Shoulder stand	OIM	4.71 (± 2.18)
	DDM	4.55 (± 1.76)
Soccer	OIM	5.36 (± 1.66)
	DDM	4.00 (± 1.65)
Vortex	OIM	4.90 (± 1.36)
	DDM	5.30 (± 1.44)
Step	OIM	4.68 (± 1.43)
	DDM	4.15 (± 1.56)

The table shows the overall scores that the experts assigned to the actions (question Q5). OIM = Observational-Imitative Method of instruction; DDM = descriptive-directive method of instruction; SD = Standard Deviation

### Soccer action

**Fig 3:**
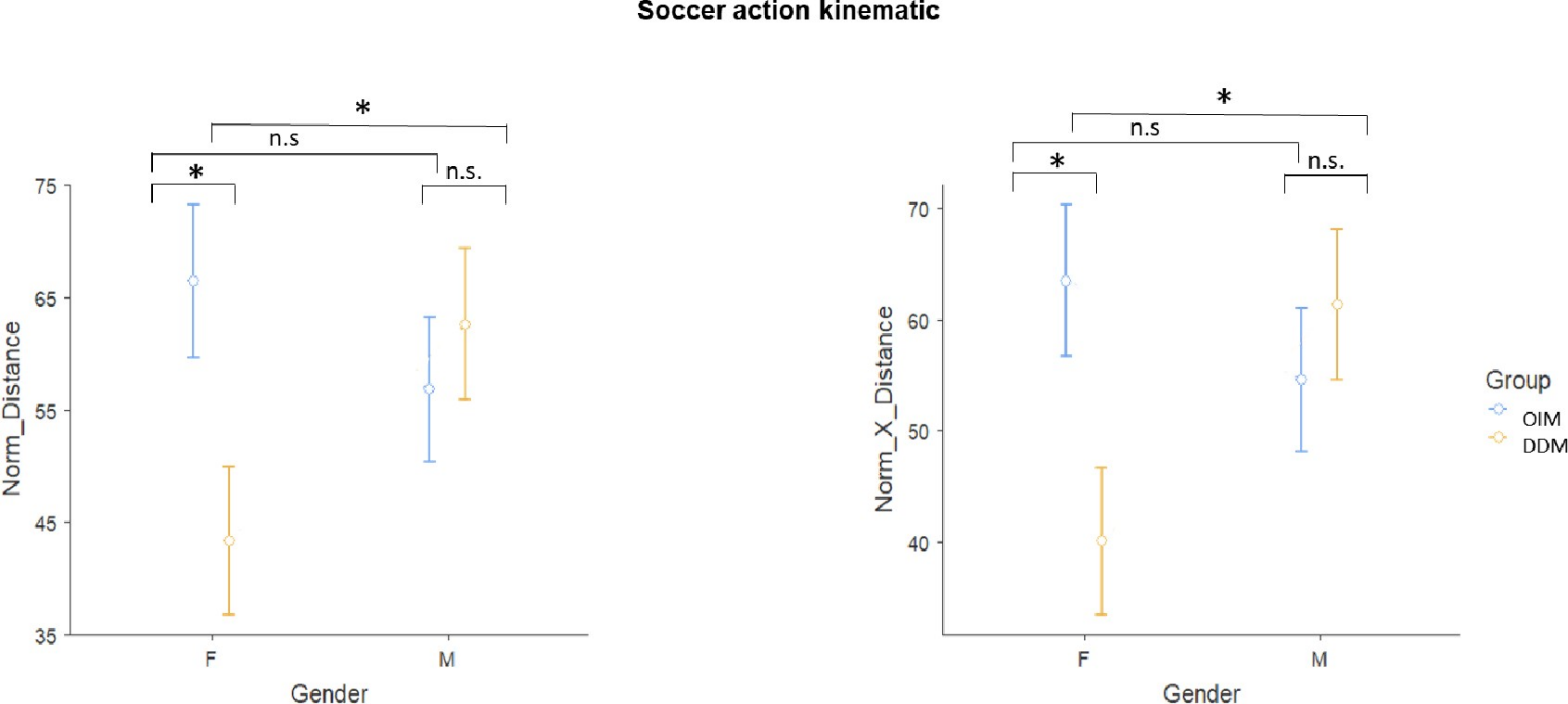
Soccer action Kinematic. Group*Gender effect related to the Norm_ Distance Kinematic parameter (left graphic) and Norm_X_Distance Kinematic parameter (right gaphic). OIM = observational-imitative method; DDM = descriptive-directive method; F = female; M = male; * = p<0.05; ** = p<0.01 and n.s = no statistical significance.

Distance and velocity parameters were considered for this action. Mixed repeated measure ANOVAs performed on each kinematic parameter showed a significant main effect of group (F_(1,31)_ = 5.13; p = 0.031, η^2^ = 0.142, OIM = 144 ±59; DDM = 114 ±35), only in the Norm_Path (see Fig 3). A significant group * gender interaction was also found for Norm_distance (F_(1,31)_ = 4.74; p = 0.037, η^2^ = 0.133) and Norm_X_Distance (F_(1,31)_ = 5.11; p = 0.031, η^2^ = 0.142). Post-hoc comparisons showed a tendency towards a difference between OIM and DDM in females only (p = 0.08 in both kinematic parameters). Females in the DDM group showed the lowest values (OIM = 63.461 ±33.5; DDM = 40.098 ±14.866). Male presented no differences (OIM = 54.590 ±19.031; DDM = 61.325 ±25.594). Statistics for all the other kinematic parameters are reported in Table 1. Once again, the experts assigned higher scores to the OIM group (5.4±1.7) then to the DDM group (4.0±1.7; Table 2).

### Vortex howler throw

For this action, after a short run-up, the participants threw the tool parabolically. Both distance and velocity parameters were considered useful in describing this action.

**Fig 4:**
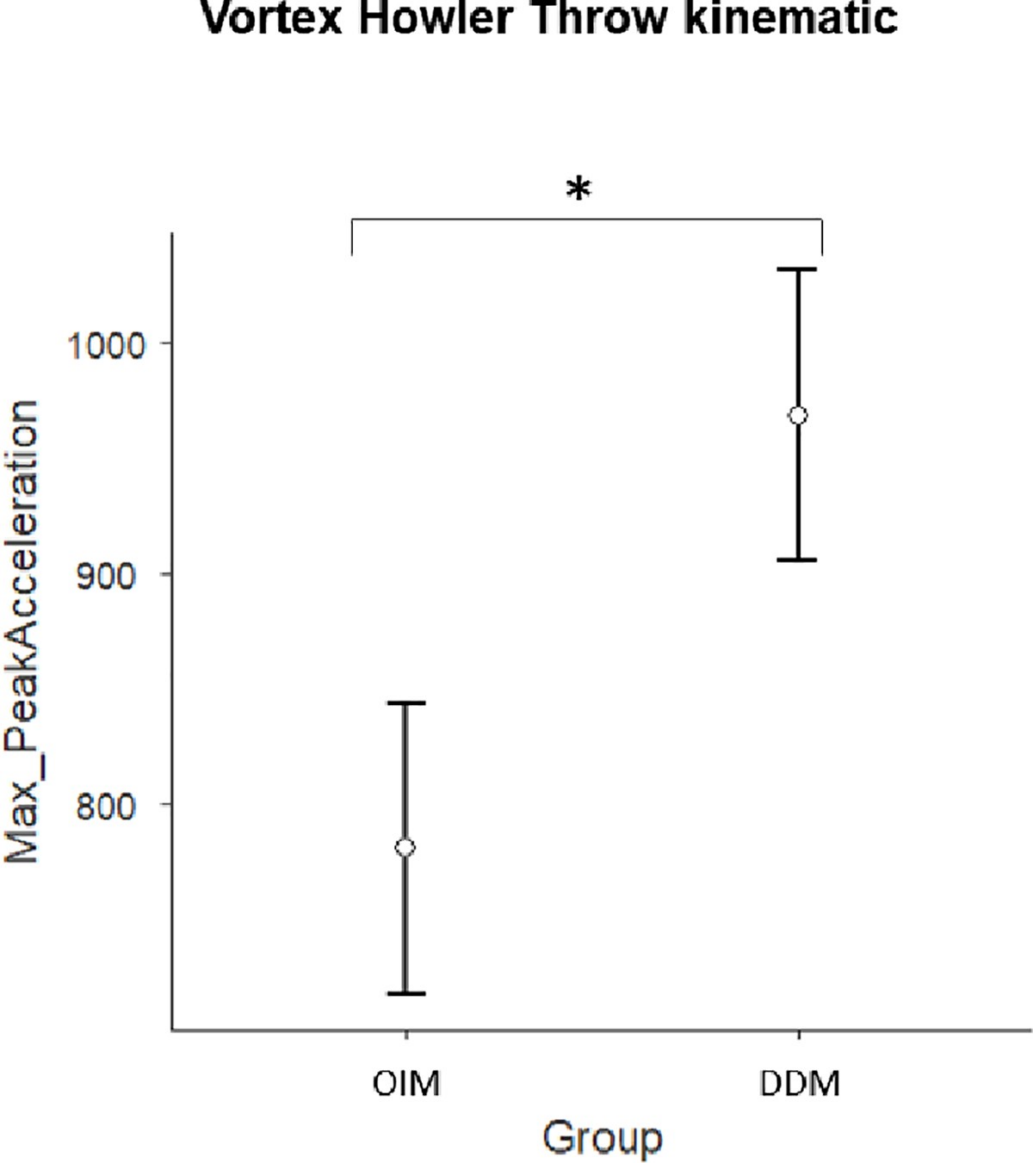
Vortex howler throw Kinematic. Main Group effect related to the Max_PeakAcceleration Kinematic parameter. OIM = observational-imitative method; DDM = descriptive-directive method; * = p<0.0.

Only Max_PeakAcceleration showed a significant group effect (F_(1,32)_ = 4.44; p = 0.043, η^2^ = 0.122). Specifically, the OIM group decelerated less than the DDM group (788 ± 266 and 960 ± 316 respectively, Fig 4). Distance parameters showed an improvement in the distance walked. Statistics for all the other kinematic parameters are reported in Table 1. The experts assigned higher scores to the DDM group (5.3±1.4) than to the OIM group (4.9±1.4; Table 2).

### Step action

No statistically significant effects were found for the step action.

## Discussion

The purpose of this pilot study was to investigate the effectiveness of two different methods of instruction (OIM and DDM) on the performance of a new motor sequence by 8-9-year-old children.

Specific sport sequences were identified in order to verify if one method of instruction was more effective than the other in terms of learning new motor actions that required the assembly of various basic motor skills.

Children’s’ performances were video-recorded and 2D kinematics then analyzed. Each video was also evaluated by blind sports experts, who assigned a score of 1 to 10 for each sport action.

For the *shoulder stand action*, Norm_Y_Distance represents the Euclidian distances between the starting and ending points in the Y dimension corresponding to the leg extension. Shoulder stand is an inverted pose, in which the body rests on one’s shoulders with legs straight. It requires children to bring their legs up and keep them straight. Consequently, Norm_Y_Distance represents the parameter that best describes the correct execution of the exercise. In fact, higher Norm_Y_Distance values represent a higher leg extension. The higher values in the Norm_Y_Distance kinematic parameter in the OIM compared to the DDM group suggests that observation of a model performing a sport action facilitates its subsequent execution. These findings are in line with previous studies investigating the effect of model observation on motor learning [55]. The observation of an expert athlete might support recognition of the main motor patterns, improving a child’s skill to a greater extent compared to children who received verbal feedback [1,45]. We also observed that in the DDM group, the Norm_Y_Distance values were lower for the first repetition of the exercise compared to the OIM group, but the performance of the DDM group improved with repetition of the exercise. This interesting result suggests that in the absence of a model, the DDM method can be effective, but it requires several repetitions of the action. This finding was confirmed by the experts, who assigned a slightly higher score to the OIM compared to DDM group.

For the *soccer action*, children threw the ball with both hands and then kicked it with their right instep after the first rebound. The correct execution of this action reflects the ability of the children to perform the motor sequence and to respect the assigned distances for hitting the ball. Thus, according to the experts, both distance and velocity parameters were reliable indexes of a correct execution of the action. Overall, the performance of the OIM group received higher scores from experts than the DDM group. Nevertheless, kinematic results suggest that the method of instruction was irrelevant for male performance. Otherwise, females in the DDM group seem to not have performed the action respecting the assigned distances. This gender difference may reflect the level of experience in this particular sport (cultural effect, [56]). In fact, in Italy, soccer is the most practiced sport (1,062,294 athletes, [57]). The FIGC (the Italian soccer federation) report (referred to 2015–16) reveals that in the juvenile sector (5–15 years), 664.729 boys and 8.826 girls are playing [58]. In light of this cultural factor, the data could reflect the fact that when an action is well-known, at least in its basic movements (such as in the case of boys who are more used to playing soccer and kicking a ball), both methods (OIM and DDM) are equally effective.

For the *Vortex Howler Throw*, after a short run-up, participants had to throw the tool parabolically. According to the experts, similarly to the soccer action, both distance and velocity parameters were reliable indexes of a correct execution of the action. We found a group difference in Max_PeakAcceleration values. The overall judgments of the experts resulted in the DDM group receiving higher scores then the OIM group. It is likely that this throwing gesture is familiar and common to all children at this age. As such, the DDM group benefited from the verbal instructions, which provided more precise information on how the vortex had to be taken and thrown. It is likely that such a method of instruction helped the children to focus on the key elements of an already familiar movement, making them more accurate than those in the OIM group. Specifically, for the vortex action, the OIM resulted in children being more focused on the overall goal (to throw the object), following their natural predisposition to imitate the goal of an action [41]. In contrast, the DDM method of instruction drove the focus of attention towards specific motor aspects of the sequence (i.e. how to grasp the object, how to adjust the shoulder and arm during the charge, etc.), facilitating execution of the action [35].

*Step action*: No significant differences were found for the step action. This action requires the execution of numerous sub-goals (take a short run-up, jump by placing only one foot on the step, rotate the body 180°, etc.). Clearly, children understood what the overall goal of the action was, but the action complexity (i.e. the level of coordination required to run, jump on one leg rotating the body) appeared very high, invalidating the performance independently of the instruction method. This was confirmed by experts who gave children the lowest scores for this action compared to all other actions.

These results could be interpreted in terms of a learning strategy that has been strongly demonstrated in studies involving very young children, named motor adaptation. Motor adaptation is a well-established movement in response to a predictable change [59,60]. During motor adaptation, the nervous system uses error information to improve future movements [61]. It is therefore likely that in a challenging motor task children focus more on the general goal of an action, and tend to improve their performance based on error-driven learning. Nevertheless, adapted motor patterns, which are stored and recalled, need more than a single training session to arise [62]. Clearly, further investigations are needed to understand the mechanisms of learning extremely complex actions, and of the role played by motor adaptation, based on the analysis of number and type of errors for each trial. It may be helpful to increase the number of repetitions used here in order to compare DDM and OIM effectiveness, and/or to assess motor learning in the follow-up assessment.

The results of this study suggest that when the motor sequence to be performed is new and does not belong to the motor repertoire of a child, the OIM instruction is more effective than DDM. This is in line with the literature claiming that during early development in particular, human learning is often strictly related to behavioral observation and imitation learning [41]. In this case, movement demonstration would play a critical role in recognizing and coding the action-goal to be imitated [41,63,64]. The natural tendency to imitate has been widely reported in human and nonhuman species [65–68], demonstrating it to be an evolutionary ancient cognitive adaptation to complex social societies. Imitation is also a pivotal skill in early development, through which children learn new skills and engage in social interactions with others [69,70], in addition to playing a key role in sport skills acquisition [71]. This is especially true in physical education [72]. Furthermore, the natural tendency of our species to automatically imitate observed actions indicates that the capacity to activate motor programs congruent and compatible with those observed relies on biologically-rooted mechanisms, which are already functional during the early stages of development [73]. This point should be taken into account in educational programs aimed at teaching new motor skills to children.

From a neurobiological perspective, imitative ability relies on a widely documented mirror mechanism [8–10,12,14,18,74–81]. Due to the overlapping neural substrate for action observation and execution, high level cognitive functions have now been attributed to mirror mechanisms, including imitation (e.g. [82]. ‘Mirror neurons’ implement an internal model for movement planning, control, and learning, thus an observed act is transformed into motor codes [83,84]. In the context of sport actions, this activation of motor programs may allow an athlete to infer the intentions of another person performing an action. During acquisition of a new motor task, observational learning allows learners to determine which the more efficient execution strategy is. However, here we also observed that (at least as far as the shoulder stand action), the repetition of the exercise permitted the DDM group to perform as well as the OIM group.

This result are in line with previous findings suggesting that model observation facilitates early stages of skill learning conveying motion information essential for the assembly of a novel or unfamiliar motor sequence [33,85]. It also stimulates a number of interesting considerations. The most important aspect of demonstration is that observation of a model benefits the understanding of motor skills by the novice prior to actual performance [32,86]. Nevertheless, another important variable must also be taken into account: the skill level of the model. A model who is not sufficiently experienced or, on the contrary, has skills far superior to the observer, may not be of benefit to inexperienced individuals [87,88]. In such cases, verbal instruction can be an equally valid training method as observation, but only if the action is repeated numerous times.

Finally, when a task is quite simple or when the child has prior knowledge of the movement to be performed (such as the vortex action, which is a simple object launch), the verbal instruction could help children focus their attention on specific motor patterns of the action.

### Final considerations

The overall results of this research have important implications for educational strategies aimed at teaching new motor skills to children. More specifically, they suggest that: a) physical education teachers and sport trainers should give priority to the demonstration of exercises (live or video) when the sport action is performed for the first time. The OIM method appears particularly effective if children do not have in-depth experience of the motor patterns underlying execution of an action; b) in absence of a model, a verbal description of a sport action needs to be accompanied by sufficient practice (i.e. the execution of the action should be repeated many times). However, in everyday life, children are continuously exposed to actions they must learn. Several competitive processes could be at stake, which may interact with the mechanisms of imitation. For example, the demonstration of an action performed perfectly may ‘intimidate’ the learner instead of informing them, especially if subjects are beginners in a specific sport [71], as well as an expert model rather than the videotaped of one's own exercise may improve performance because subjects want to perform like the model performing a perfect execution [89]. Since feedback and instructions play a powerful role in guiding the performance future studies should be done in different sport settings with models at different levels of expertise and with actions of different complexity.

### Limits

The most obvious limitation of this research is the small sample size, a limitation that prevented a clear generalized statement about the role played by OIM or DDM in a child’s performance. Nevertheless, we think that these preliminary results are encouraging, and should be confirmed and explored in future research. This study was also limited by the duration of the research, which was relatively short; consequently, the performance of the participants was observed over a relatively short period of time. Furthermore, some other critical aspects warrant further investigations. The first one concerns the type of model (novice or expert) demonstrating the “correct” performance to be imitated. Expert model observation provided the observer with an accurate template for performing the task. However, watching a model learning a new sport action could help the observer associate different movement patterns with different outcomes (based on error-driven learning). Notably, observing a novice model’s (e.g. peer) less accurate performance, compared to an expert [90], induces the observer to adopt different movement strategies [91]. For these reasons, future studies could investigate the effectiveness of the OIM method on training with peers.

Additionally, we did not take into account the potential limitations presented by differences in working memory processing. This might interfere with a child’s capacity to accurately reproduce an observed action, particularly when the action to be performed is composed of several motor acts. Other studies have shown that the learning a new action involves the construction and modification of an internal model. However, motor memory, which allows the brain to implicitly predict the behavior of the action to be performed based on such internal models, can decay over time. Type of training and schedule of repetitions could affect the decay rate [92].

Finally, no feedback was given to the children here concerning the outcome of their performance [93]. The effects of feedback on the efficacy of OIM and DDM methods to teach new sport actions is another aspect worth further investigation.

## Supporting information

S1 Fig Frames video selected for the feasibility study: (A) Participants threw a ball against the wall and grabbed it. This exercise was performed standing on one-foot (B) Participants grasped and moved a ball from one support to another. They stood on one foot maintaining the balance on an unstable platform.
(TIF)

S2 Fig ANOVA performed on frequencies of correct trials transformed in arcsine values (ordinal axes). In abscissa axes experimental conditions are reported (OIM = observational-imitative method; DDM = descriptive-directive method). Error bars represent SE (standard errors of the means).
(TIF)

S1 Database (XLSX)

S1 Materials (DOCX)
