# Supplementary figures and images for: Learning new sport actions: Pilot study to investigate the imitative and the verbal instructive teaching methods in motor education
Source: PLoS One. 2020 Aug 14;15(8):e0237697. doi: 10.1371/journal.pone.0237697 (PMC7428179; doi:10.1371/journal.pone.0237697)

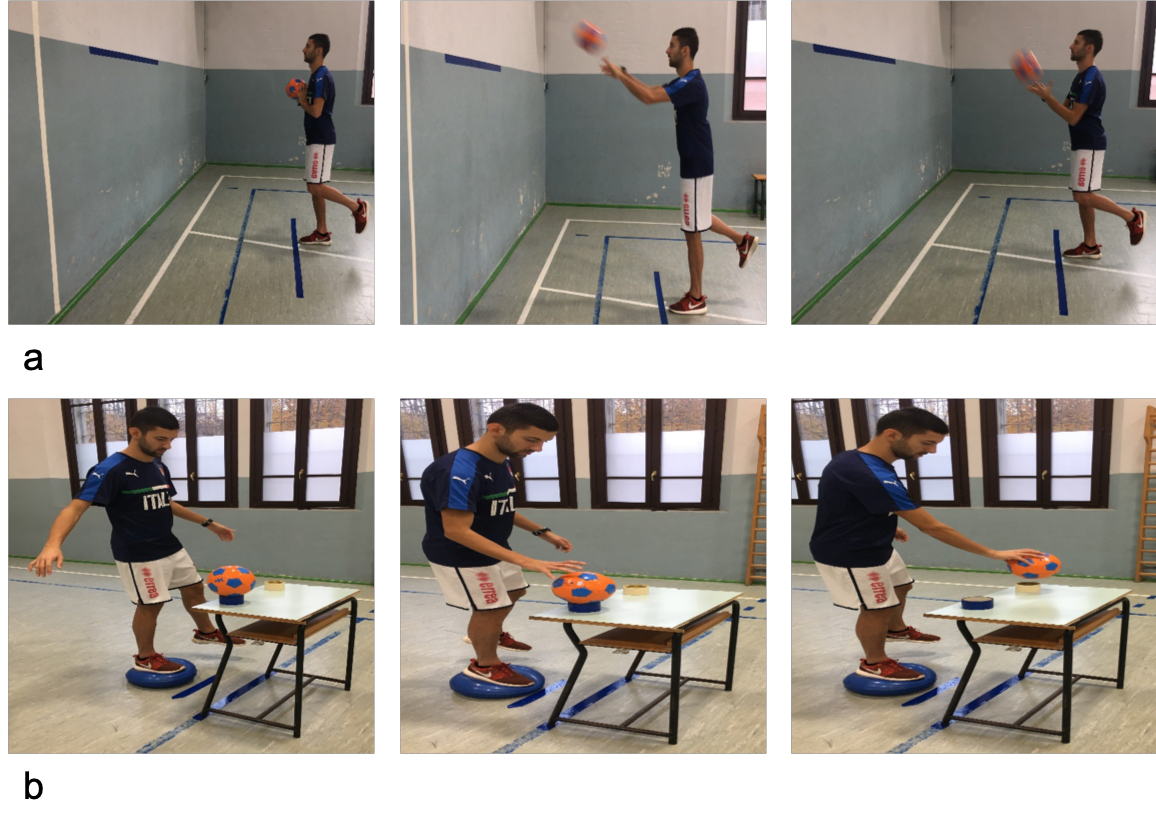

Supplement: S1 Fig — Frames video selected for the feasibility study: (A) Participants threw a ball against the wall and grabbed it. This exercise was performed standing on one-foot (B) Participants grasped and moved a ball from one support to another. They stood on one foot maintaining the balance on an unstable platform. (TIF) [file pone.0237697.s001.tif]

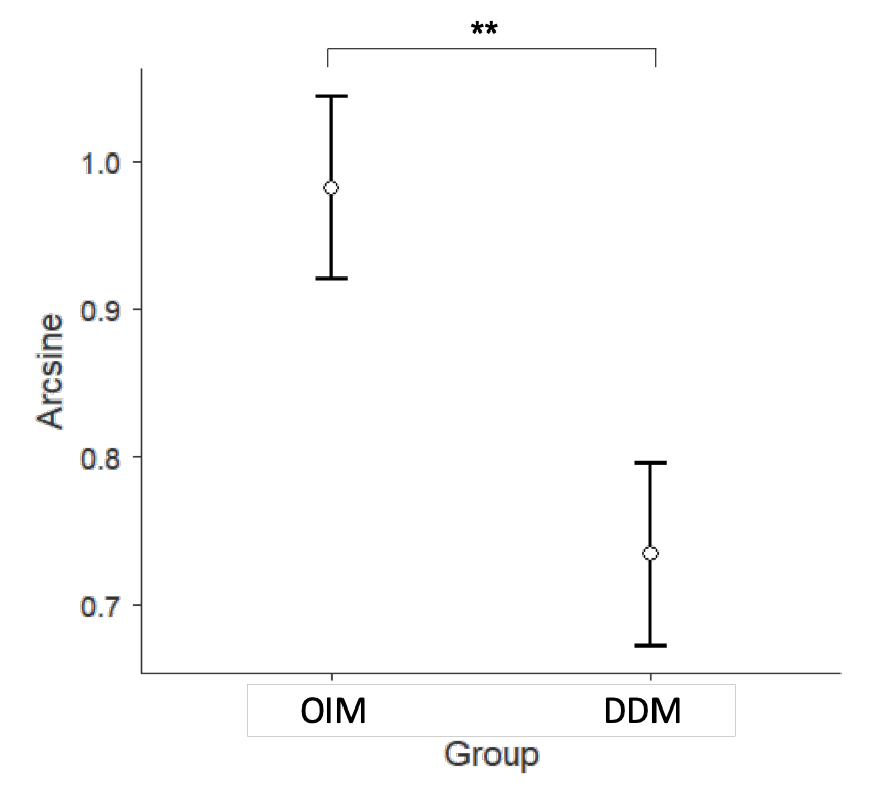

Supplement: S2 Fig — In abscissa axes experimental conditions are reported (OIM = observational-imitative method; DDM = descriptive-directive method). Error bars represent SE (standard errors of the means). (TIF) [file pone.0237697.s002.tif]
