# Supplementary material for: Learning new sport actions: Pilot study to investigate the imitative and the verbal instructive teaching methods in motor education
Source: PLoS One. 2020 Aug 14;15(8):e0237697. doi: 10.1371/journal.pone.0237697 (PMC7428179; doi:10.1371/journal.pone.0237697)
Supplement: S1 Materials — (DOCX) [file pone.0237697.s004.docx]

## **FEASIBILITY STUDY**

*Participants*

20 healthy children (8 males and 12 female) aged between 8 and 9 participated in the feasibility study (M_age_=8; SD=0.5). Exclusion criteria were: (1) the achievement of a score <=70 percentile (M=30.6; SD=3.13) at the Test Coloured Progressive Matrices, CPM (a test used for the evaluation of cognitive functions, general components of fluid intelligence which allows the investigation of the capacity of problem- solving; [1]); (2) any orthopedic or neurological problems that would interfere with the ability to perform a coordinated arm movement; (3) left dominant hand.

*Procedure*

The feasibility study aimed to assess whether children aged 8-9 years old are able to perform exercises that required visual-motor and proprioceptive abilities, following the instructions provided in the two methods: OIM and DDM. The children, equally distributed by gender and number, were randomly assigned to two groups (OIM and DDM) each composed of 10 children. Physical teachers and experts of the Giocampus reality in Parma (Italy) assisted us with the selection of the critical skills and their essential components. The following actions have been selected: (1) Participants threw a ball against the wall and grabbed it. This exercise was performed standing on one foot (Fig 1A). (2) Participants grasped and moved a ball from one support to another while balancing themselves on one foot on an unstable platform (Fig 1B). The OIM group observed three minutes of videos in which an athlete performed the entire action correctly. No verbal explanation was given. The DDM group received only verbal instructions (see Supplementary Materials) for three minutes. The verbal instruction for the action one of the feasibility study was: “throw the ball against the wall and grab it”. About the second action it was “grasp the ball on the frontal table and move it from the first to the second box”. Three frames for each action were associated with verbal explanations (Fig 1).

OIM and DDM groups were then required to repeat each action 10 times consecutively (trial 1 to 10).

**S1 Fig**. Frames video selected for the feasibility study: (A) Participants threw a ball against the wall and grabbed it. This exercise was performed standing on one-foot (B) Participants grasped and moved a ball from one support to another. They stood on one foot maintaining the balance on an unstable platform.

A physical teacher reported the correct or incorrect execution of participants in each trial on a score sheet. This scoring procedure was applied to both tasks.

**Statistical analysis**

A mixed repeated measure ANOVA was performed on frequencies of correct trials (index of exercise accuracy) considering the group factor (OIM vs DDM) and the gender (female vs male) variables as between factors and task (1: throw the ball and grasp it vs 2: balance and move) as within factor. Frequencies of correct trials were arcsine transformed prior to the analysis; values ranged from a minimum of zero to a perfect score of 1.57 (which is the arcsine of 1), the value obtained for a correct performance [2]. When the sphericity assumption was violated, Greenhouse–Geisser degrees of freedom corrections were applied. The probability value was set at p < 0.05 for all analyses. Newman-Keuls post-hoc tests followed the ANOVA.

**Results**

The results showed a statistically significant difference between the two type of instruction (F(1,16)=7.97, p=0.012, η2=0.33) (Fig 2). The OIM instruction induced better performance than the DDM one.

The interaction among instruction * gender * task was also significant (F(1,16)=4.53, p=0.049, η2=0.22). Specifically, in the male group, children belonging to the DDM group performed higher errors compared to OIM group in the second task only (p=0.049). No other statistical differences were found.

**S2 Fig**. ANOVA performed on frequencies of correct trials transformed in arcsine values (ordinal axes). In abscissa axes experimental conditions are reported (OIM = observational-imitative method; DDM=descriptive-directive method). Error bars represent SE (standard errors of the means).

**References**

1. Raven JC. Progressive matrices 1938: serie A,B,C,D,E: manuale di istruzioni. Firenze: Organizzazioni speciali; 1982.

2. Wagner HL. On measuring performance in category judgment studies on nonverbal behavior. J Nonverbal Behav. 1993;17: 3–28. doi:10.1007/BF00987006

| **Descriptives** | | | | | | | |
| --- | --- | --- | --- | --- | --- | --- | --- |
|  |  |  |  |  |  |  |  |
|  | | **Group** | | **Task1** | | **Task2** | |
| Mean |  | OIM |  | 1.047 |  | 0.912 |  |
|  |  | DDM |  | 0.735 |  | 0.737 |  |
| Standard deviation |  | OIM |  | 0.227 |  | 0.227 |  |
|  |  | MDD |  | 0.327 |  | 0.264 |  |
|  | | | | | | | |

**10-POINTS LIKERT QUESTIONNAIRE**

In the table the 10-points Likert questionnaire are reported. The answer to each question were expressed with respect to a numeric rating scale in which the score of 0 represents a judgment of "Absolute NOT correctness" and 10 a judgment of “Absolute Correctness”.

| **Sport Action** | **Qualitative Judgement Question** | | | | | |
| --- | --- | --- | --- | --- | --- | --- |
|  |  |  | | | | |
| Shoulder Stand | |  | | | | |
|  | Q1 | Was the back rolling correctly performed on the axis? | | | | |
|  | Q2 | Were the hands correctly carried to the sides with the elbows tight to the body? | | | | |
|  | Q3 | Was the vertical position of the candle achieved? | | | | |
|  | Q4 | Was the candle position correctly maintained? | | | | |
|  | Q5 | How do you evaluate the overall correctness of the sport gesture? | | | | |
|  |  |  | | | | |
| Soccer Action | |  | | | | |
|  | Q1 | Was the ball correctly released? | | | | |
|  | Q2 | Was the inclination of the torso correct? | | | | |
|  | Q3 | Was the position of the arm correct? | | | | |
|  | Q4 | Did the ball correctly kick in the descending phase after the first rebound of the ball? | | | | |
|  | Q5 | How do you evaluate the overall correctness of the sport gesture? | | | | |
|  |  |  | |  | |  |
| Vortex Howler Throw | | |  | |  | |
|  | Q1 | Was the supporting leg correctly opposite to the arm that throws the vortex? | | | | |
|  | Q2 | Was the extension-flexion-extension sequence of the arm correct? | | | | |
|  | Q3 | Was the rotation correctly performed? | | | | |
|  | Q4 | Was the forward direction on the throw correctly performed? | | | | |
|  | Q5 | How do you evaluate the overall correctness of the sport gesture? | | | | |
|  |  |  | | | | |
| Step Action | |  | | | | |
|  | Q1 | Was the run correctly adapted to the distance? | | | | |
|  | Q2 | Was the rotation correctly performed? | | | | |
|  | Q3 | Was the arrival in bipodalic position? | | | | |
|  | Q4 | Was the arrival in balance? | | | | |
|  | Q5 | How do you evaluate the overall correctness of the sport gesture? | | | | |
|  |  |  | | | | |
